# Hypoglycemic Effects of Three Medicinal Plants in Experimental Diabetes: Inhibition of Rat Intestinal *α*-glucosidase and Enhanced Pancreatic *Insulin* and Cardiac *Glut-4* mRNAs Expression

**Published:** 2013

**Authors:** Leila Moradabadi, Shideh Montasser Kouhsari, Mohammad Fehresti Sani

**Affiliations:** *Department of Cellular and Molecular Biology, School of Biology, University College of Science, University of Tehran, P.O. Box 14155-6455, Tehran, Iran. *

**Keywords:** Diabetes, Glut-4, Insulin, OGTT, PBG

## Abstract

Garlic (*Allium sativum *L., Alliaceae), Persian shallot (*Allium ascalonicum *L., Alliaceae ) and Sage (*Salvia officinalis *L., Lamiaceae) are believed to have hypoglycemic properties and have been used traditionally as antidiabetic herbal medicines in Iran. In this study, diabetes was induced by subcutaneous injection of alloxan monohydrate (100 mg kg^−1^) to male Wistar rats. Antidiabetic effects of methanolic extracts of the above mentioned three plants on alloxan-diabetic rats was investigated in comparison with the effects of antidiabetic drugs such as acarbose, glibenclamide and metformin by measuring postprandial blood glucose (PBG), oral glucose tolerance test (OGTT), inhibition of rat intestinal *α*-glucosidase enzymes activities and pancreatic *Insulin *and cardiac *Glut-4 *mRNAs expression. In short term period, hypoglycemic effects of *A. sativum *and *A. ascalonicum *showed significant reduction of PBG similar to glibenclamide (5 mg kg^−1^ bw) while *S. officinalis *significantly reduced PBG similar to acarbose (20 mg kg^−1^ bw). After 3 weeks of treatment by methanolic plant extracts, significant chronic decrease in the PBG was observed similar to metformin (100 mg kg^−1^ bw). For OGTT, *S. officinalis *reduced PBG in a similar way as acarbose (20 mg kg^−1^ bw). Intestinal sucrase and maltase activities were inhibited significantly by *A. sativum*, *A. ascalonicum *and *S. officinalis*. In addition, we observed increased expression of *Insulin *and *Glut-4 *genes in diabetic rats treated with these plants extracts. Up regulation of *Insulin *and *Glut-4 *genes expression and inhibition of *α*-glucosidaseactivities are the two mechanisms that play a considerable role in hypoglycemic action of garlic, shallot and sage.

## Introduction

Diabetes mellitus (DM), a global public health problem, is now emerging as an epidemic worldwide (1). The term diabetes mellitus describes several diseases of abnormal carbohydrate metabolism that are characterized by hyperglycaemia. It is associated with a relative or absolute impairment in insulin secretion, along with varying degrees of peripheral resistance to the action of insulin. Medicinal plants provide valuable therapeutic antidiabetic agents, in traditional and modern medicine. Herbal medications have been used for a variety of ailments and in recent years, the use of natural health products as complementary or alternative approaches to existing medications is growing in popularity (2). Several ethnopharmacologial studies on medicinal plants having beneficial effects on diabetes have been reported (3). The hypoglycaemic activity of a large number of medicinal plants used in traditionally has been evaluated and confirmed in different animal models (4-6). 

Garlic (*Allium sativum *L., Alliaceae) has been used since ancient times as a folk medicine in diabetes in Iran (7-8), India (9), and Europe (10). Persian shallot (*Allium ascalonicum *L.*, *Alliaceae) is used traditionally as a hypoglycemic agent in Asia (11-12). These plants have been cultivated in all over Iran for their characteristic flavors and medicinal properties (8). Multiple biological effects of garlic and Persian shallot are related to the presence of thiosulfinates, volatile sulfur compounds that are typical of the Allium species, which are also responsible for their characteristic pungent aroma and taste (13). Sage (*Salvia officinalis *L., Lamiaceae) has wide range of biological activities (14-15) and especially its hypoglycemic effects in Iranian folk medicine due to its flavonoids such as kaempferol, luteolin and quercetin are reported (16-21). Sage antioxidant properties are attributed mainly to the presence of carnosic acid and rosmarinic acid (22).

Garlic, Persian shallot and sage are used in traditional medicine in Iran for diabetes; however, biochemical and molecular basis of their antidiabetic effects remain unclear. The purpose of the present study was to examine the influence of oral administration of garlic and Persian shallot bulbs and sage leaves methanolic extracts ( ASE, AAE and SOE respectively) on the levels of blood glucose, oral glucose tolerance, Insulin and *Glut-4 *genes expression and intestine alpha glucosidase enzymes activities in alloxan-induced diabetic Wistar rats.

## Experimental

*Chemicals and reagents*

Glibenclamide, metformin and acarbose were purchased from Tehran Chemistry (Iran), alloxan mono hydrate was purchased from Pharmacia & Upjohn (USA), DEPC water, Taq polymerase and RNA extraction kit were obtained from CinnaGen (Tehran, Iran); DNase I, RNase free kit were purchased from Fermentas (Ontario, Canada), RT kit and Primers from Bioneer (Daejon, Korea), dNTPs from BioFlux (Tokyo, Japan). All other chemicals and solvents were of the highest commercial grade from Merck (KGaA, Germany) or from Sigma (St Louis, MO, USA).

*Plants materials*

Plants materials used in this study consisted of the bulbs of *Allium sativum *L. (Alliaceae)*, Allium ascalonicum *L. (Alliaceae) and the leaves of *Salvia officinalis *L. (lamiaceae). Garlic plants were collected from Caspian sea region in the north of Iran. Persian shallots were obtained from Alborz mountain in the north of Tehran and sage plants were collected from northwest of Iran. They were authenticated by Professor Ahmad Qahraman and voucher specimen as follows: *Allium satium *L., 35842, *Allium ascalonicum *L.,35351 and *Salvia officinalis *L.,37221. They were deposited at the herbarium of University of Tehran, Tehran, Iran.

*Preparation of Allium sativum L. (ASE), Allium ascalonicum L. (AAE) and Salvia officinalis L. (SOE) methanolic extracts*

Dried and ground bulbs of garlic and Persian shallot (about 200 g) and leaves of sage were submitted to extraction with 300 mL methanol (80%) in a Soxhlet apparatus for 72 h (23). After extraction, the solvent was filtered and then evaporated by Rotavapor. The percentage yields based on the dried starting materials were 20% for garlic, 17% for shallot and 23% for sage. The mehtanolic extracts were stored in the dark at 4°C until used for experiments. 

*Animals*

Male Wistar rats, weighing 200–250 g (Pasteur Institute, Tehran, Iran) were used in this study. Animals were housed six per standard rat cage, in a room with a 12:12 h light/dark cycle (lights on 07:00 h) and controlled temperature (22 ± 1 °C). Commercial rodent pellets and tap water were available *ad libitum*. They were allowed to adapt to the laboratory conditions for one week before beginning of the study. There were six rats per group in each experiment. The procedures were performed in accordance with institutional guidelines for animal care and use. 

*Preparation of alloxan-induced diabetic Wistar rats*

Diabetes was induced in overnight fasted rats by subcutaneous injection of alloxan monohydrate (100 mg kg^−1^), dissolved in citrate buffer (pH = 4.5), according to a previously described method (24-25). After one week of administration, survived rats with marked hyperglycemia (postprandial blood glucose > 250 mg/dL) were selected and used for this study.

*Experimental design*

Rats were randomly divided into the following ten groups, each group consisting of six animals.

Group I (NC): Normal rats treated with vehicle alone; Group II (DC): Diabetic rats treated with vehicle alone; Group III (ASEa+D): Diabetic rats treated with ASE at the dose of 250 mg kg^−1^ BW; Group IV (ASEb+D): Diabetic rats treated with ASE at the dose of 500 mg kg^−1^ BW; Group V (AAEa+D): Diabetic rats treated with AAE at the dose of 250 mg kg^−1^ BW; Group VI (AAEb+D): Diabetic rats treated with AAE at the dose of 500 mg kg^−1^ BW; Group VII (SOEa+D): Diabetic rats treated with SOE at the dose of 250 mg kg^−1^ BW; Group VIII: (SOEb+D): Diabetic rats treated with SOE at the dose of 500 mg kg^−1^ BW; Group IX: (Ac+D): Diabetic rats treated with acarbose (20 mg kg^−1^ BW) in the 1st phase; Group X: (Glib+D in the1st phase or Met+D in the 2nd phase): Diabetic rats treated with glibenclamide (5 mg kg^−1^ BW) or metformin (100 mg kg^−1^ BW). 

In the first phase of this study, a single dose of each sample was administered to all rats and postprandial blood glucose levels (blood glucose level after eating a meal) were estimated in a short-term model. In this phase acarbose and glibenclamide were used as the reference drugs. After two days, Oral Glucose Tolerance Test (OGTT) was carried out for all rats and acarbose was used as the reference drug.

In the second phase of this study, two days after OGTT, one dose of samples was administered daily for 21 days (26) to all rats. In this phase metformin was used as the reference drug. Postprandial blood glucose levels were estimated in a long-term model. At the end of 21 days of treatment, rats were anesthetized by ether, then the animals were killed and the pancreases and hearts of NC, DC, ASEb+D, AAEb+D and SOEa+D groups were removed promptly for the estimation of insulin (*Ins*) and glucose transporter-4 (*Glut-4*) mRNA expression. In a separate experiment, the inhibitory effects of plants extracts on intestinal *α*-glucosidases (sucrase and maltase) were measured by an *in-vitro *method.

*Oral administration of the plant extracts*

1mL of each plant extract sample was administered orally at 11-12 a.m. using an intragastric tube.

*Estimation of hypoglycemic activity*

*Short-term experimental model*

Blood samples were obtained from the tail vein and postprandial plasma glucose levels were estimated using a glucometer (On Call Now, San Diego, USA) after 1, 3, 5, 8 and 24 h following administration of a single dose of samples to rats. The NC and DC groups were treated by the same volume of vehicle (1 mL of distilled water).

*Long-term experimental model*

During the long-term treatment period with ASE (250 and 500 mg kg^−1^ BW), AAE (250 and 500 mg kg^−1^ BW), SOE (250 mg kg^−1^ BW) and metformin (100 mg kg^−1^ BW), the levels of postprandial plasma glucose in all rats were estimated at the end of 1, 2 and 3 weeks of treatments using blood samples obtained from tail vein and a glucometer device (On Call Now, San Diego, USA).

*Measurement of ASE, AAE and SOE effects on Oral Glucose Tolerance*

NC and DC Groups received orally distilled water. ASEa+D and ASEb+D groups received orally ASE at doses of 250 and 500 mg kg^−1^ BW respectively. AAEa+D and AAEb+D groups received orally AAE at doses of 250 and 500 mg kg^−1^ BW respectively. SOEa+D group received orally SOE at dose of 250 mg kg^−1^ BW. To group Ac+D, the reference drug acarbose (20 mg kg^−1^ BW) was administrated orally. Thirty minutes later, a carbohydrate solution (equal proportion of maltose and sucrose 2 g kg^−1^ BW) was administered orally to each rat (27). PBG was determined at 0 min, just before carbohydrate solution loading, and at 30, 60 and 120 min after carbohydrate solution loading, using a glucometer (On Call Now, San Diego, USA).

*Gene expression analysis*

To investigate the mechanism of ASE, AAE and SOE antihyperglycemic action, at the end of 21 days of treatment, the animals in DC, NC, ASEb+D, AAEb+D and SOEa+D groups were analyzed for *Ins *and *Glut-4 *mRNAs expression by RT-PCR (Reverse Transcription Polymerase Chain Reaction). *β*-actin gene was used as internal control. Total RNA of pancreas and heart tissues (28) of each rat was extracted by RNX-Plus kit. In brief, after homogenization of tissue samples (1 mL per 50-100 mg tissue) with RNX-Plus kit, proteins were extracted with chloroform and total RNA was precipitated with isopropanol. The precipitated RNA was washed with 70% ethanol and resuspended in 50 μL of DEPC-treated water. Finally the DNA free RNA was prepared prior to RT-PCR using DNase I, RNase-free kit. Reverse transcription was carried out to obtain cDNA using AccuPower RT PreMix kit, 50 ng/μL template RNA and 25 ng/μL oligo dT18. The primers used were as follows: *Ins *F, 5′-TTC TTC TAC ACA CCC AAG-3′; *Ins *R, 5′-GCA GTA GTT CTC CAG TTG-3′ (155-bp); *Glut-4 *F, 5′-AGG CAC CCT TAC CCT TTT-3′; *Glut-4 *R 5′-GAC AGA AGG GCA ACA GAA GC-3′ (318-bp) and *β*-act F, 5′-AGC CAT GTA CGT AGC CAT CC-3′; *β*-act R,5′-TCT CAG CTG TGG TGG TGA AG-3′ (248-bp). For PCR reaction, 500 ng of the cDNA was added to a PCR reaction mixture consisting of 10XPCR buffer (2.5 μL), 50 mM MgCl_2_ (0.75 μL), 10 mM dNTPs (0.5 μL), 10 pM of paired primers (0.5 μL of each), 0.25 units of Taq polymerase and distilled water in a total volume of 25 μL. The reaction mixture was loaded in a PCR thermal cycler for 35 cyclic reactions. PCR products were run on 1.5% agarose gels, stained with ethidium bromide and photographed. Images of radiographs were analyzed with TotalLab v1.10 using 1D analysis.

*Inhibition assay for rat intestinal sucrase activity*

Inhibition of rat intestinal sucrase was assayed using previously reportedmethod (29-30) with a slight modification. 0.2 mL of 56 mM sucrose, as the enzyme substrate, in 0.1 M potassium phosphate buffer (pH 7, 0.2 mL) was mixed with 0.1 mL of the plant extracts in 50% aqueous dimethyl sulfoxide (DMSO). After pre-incubation at 37° C for 5 min, 0.2 mL of rat intestinal *α*-glucosidase solution prepared from intestine of normal rats (31) was added. Instead of the plant extract, 0.1 mL DMSO was used for the blank sample. After mixing thoroughly, both samples and blank test tubes were incubated at 37 °C for 15 min and then the reaction was stopped by submerging test tubes in boiling water for 4 min. The reaction mixture was passed through a basic alumina column (6 mm × 35 mm h) to eliminate phenolic or acidic compounds. The amount of liberated glucose was determined by the glucose oxidase assay using a commercial test kit. The optical density (OD) of the wells was measured at 505 nm and the inhibitory activities of plants extracts were calculated using following formula: 

Inhibitory activity (%) = 100 (1-[ODtest sample/ODcontrol] ) 

*Inhibition assay for rat intestinal maltase activity*

Inhibition of rat intestinal maltase was determined by using reported method (29, 32) with a slight modification. The assay was carried out in the same manner as the inhibition assay for rat intestinal sucrase, except for using 3.5 mM maltose, as the enzyme substrate, in 0.1 M potassium phosphate buffer (pH 7, 0.35 mL).

*Statistical analysis*

All data are presented as mean ± SD. for six animals in each group. Comparisons between groups and between time points were made by one-way analysis of variance (ANOVA) followed by Duncan’s test to analyze the difference. Differences were considered significant when P-values were less than 0.05. All statistical analyses were performed using SPSS (SPSS Inc, Chicago, USA). 

## Results

*Effects of ASE, AAE and SOE on PBG in short term treatment*

Table 1 depicts antihyperglycemic effects of two different doses of ASE, AAE and SOE at different intervals of time after a single dose oral administration of each extract, acarbose (20 mg kg−1 BW) and glibenclamide (5 mg kg−1 BW) as reference drugs. 

**Table 1:** Acute effects of ASE, AAE and SOE on postprandial blood glucose in alloxan-diabetic Wistar rats

**Groups**	**Dose**	**Postprandial blood glucose (mg/dL)**					
		**(mg kg** ^−1^ **BW)**					
		**0**	**1**	**3**	**5**	**8**	**24**
NC	-	68.2±2.3^b^	71.2±6.4^b^	64.8±2.8^b^	74.4±3.3^b^	75.2±4.5^b^	70.34±4.4^b^
DC	-	314.2±10.3^c,d^	318.7±14.4^d^	309.34±15.2	303.22±8.2	312.32±12.3	322.21±10.3
ASEa+D	250	309.53±15.7^c,d,e^	307.45±11.4^d,e^	289.41±9.4^a,b,d^	281.62±12.3^a,b^	274.82±8.3^a,b^	282.65±9.1^a,b^
ASEb+D	500	311.4±12.5^c,d,e^	309.61±8.9^d,e^	287.34±11.8^a,b,d^	259.3±12.6^a,b,d^	246.52±9.6^a,b^	241.66±9.2^a,b^
AAEa+D	250	308.44±14.4^c,d,e^	316.4±13.1^d,e^	311.64±8.2^e^	285.53±10.3^a,b^	287.39±8.51^a,b^	283.3±14.2^a,b^
AAEb+D	500	311.24±13.2^c,d,e^	304.81±7.1^d,e^	291.5±8.9^a,b,d^	273.24±11.7^a,b^	270.41±10.7^a,b^	274.23±7.4^a,b,c^
SOEa+D	250	314.67±11.1^c,d,e^	289.6±10^a,b^	269.33±9.2^a,b,c^	290.33±15.5^a^	292.3±9.2^a,b^	271±11.5^a,b,c^
SOEb+D	500	320.66±19.5^c,d,e^	318.66±14^d,e^	298.33±13.5^a,d,e^	310.5±9^e^	320±15.13^e^	324.3±14.2^e^
Ac+D	20	318.2±10.2^c,d,e^	265.2±7^a,b^	256.2±7.3^a,b^	186.2±14.8^a,b^	199.4±7^a,b^	266.8±7.8^a,b^
Glib+D	5	312.4±14.3^c,d,e^	303.11±7.9^e^	291.6±12.8^a,b^	245.73±9.1^a,b^	174.32±9.6^a,b^	129.44±8.2^a,b^

NC: normal control, DC: diabetic control, ASEa+D and ASEb+D: diabetic rats treated with 250 and 500 mg kg^−1 ^BW of *Allium sativum *bulbs methanolic extracts respectively, AAEa+D and AAEb+D: diabetic rats treated with 250 and 500 mg kg^−1 ^BW of *Allium ascalonicum *bulbs methanolic extracts respectively, SOEa+D and SOEb+D: diabetic rats treated with 250 and 500 mg kg^−1 ^BW of *Salvia officinalis *leaves methanolic extracts respectively, Ac+D: diabetic rats treated with acarbose, Glib+D: diabetic rats treated with glibenclamide. Each value is the mean ± SD. of ten separate experiments. ^a ^p < 0.05 vs. Time 0, ^b^ p < 0.05 vs. DC, ^c^ p > 0.1 vs. Ac+D, ^d^ p > 0.1 vs. Glib+D, ^e^p > 0.06 vs. DC

ASE (250 and 500 mg kg^−1^ BW ) and AAE (500 mg kg^−1^ BW) at 3h reduced significantly PBG, 6%, 7% and 5.7% respectively (p < 0.05 vs. DC) and decreased gradually PBG till 24 h (p < 0.05 vs. DC), similar to glibenclamide. SOE (250 mg kg^−1^ BW) reduced significantly PBG at 1 h 9% and 3h 13% (p < 0.05 as compared with DC). Then gradual rise occurred in PBG till 8 h. Moreover, at 24 h, PBG is reduced 14% (p < 0.05 as compared with Time 0). The effects of SOE (250 mg kg^−1^ BW) at 3h and 24h were similar to that of acarbose (p > 0.1). SOE (500 mgkg^−1^ BW) reduced significantly PBG (p < 0.05 as compared with Time 0) at 3h but it showed no notable difference when compared with DC (p > 0.06), thus this dose was eliminated in following experiments. At 24 h, ASE (500 mg kg^−1^ BW) and glibenclamide (5 mg kg^−1^ BW) produced major fall in PBG 25% and 60% (p < 0.05 vs. DC) respectively, but AAE (500 mg kg^−1^ BW) and acarbose (20 mg kg^−1^ BW) showed noticeable decrease in PBG at 8 h, 13% and 36% (p < 0.05 vs. DC) respectively.

*Effects of ASE, AAE and SOE on oral Glucose tolerance*

Table 2 shows that PBG concentrations increased 30 min after oral administration of carbohydrate solution, in all groups and decreased subsequently. The increase in PBG levels, compared to DC group, diminished significantly in ASEa and ASEb groups at 30 min by 10% and 9%, at 60 min by 9 % and 7% and at 120 min by 11% and 13% and in AAEa and AAEb groups at 30 min by 12 % and 11 %, at 60 min by 6 % and 8 % and at 120 min by 9 % and 12 % (p < 0.05). SOEa group showed diminution in PBG levels at 30 min by 17 %, at 60 min by 32 % and at 120 min by 13 % (p < 0.05). These results show that the reduction of PBG by SOE at dose of 250 mg/kg BW, at 60 min was 32 % (p < 0.05) and this effect is comparable with 36 % reduction of PBG by acarbose (p > 0.1). 

**Table 2:** Effects of ASE , AAE and SOE on oral glucose tolerance test in alloxan-diabetic rats. The plants extracts and the reference drug was administered to 16 h fasted rats, and 30 min later all groups received a carbohydrate solution (maltose and sucrose each 1 g/kg BW) by gastric intubations. Postprandial blood glucose was monitored at 0, 30, 60 and 120 min after carbohydrate solution administration

**Groups**	**Dose** **(mgkg** ^−1^ **BW)**	**Blood glucose (mg/dL)**			
		**minute after a single dose administration of extracts and drug**			
		**0**	**30**	**60**	**120**
NC	-	70.32±3.2^b^	139.23±7.3^a,b^	119.38±5.1^a,b^	87.76±4.3^a,b^
DC	-	186.74±7.4	329.56±14.8^a^	288.72±11.7^a^	254.68±13.3^a^
ASEa+D	250	189.72±15.5	295.41±9.1^a,b^	261.8±7.9^a,b^	225.72±10.3^a,b^
ASEb+D	500	194.37±13.2	298.63±8.2^a,b^	267.73±12.2^a,b^	219.86±14.1^a,b^
AAEa+D	250	192.25±10.1	289.92±13^a,b^	270.43±8.2^a,b^	231.3±11.6^a,b^
AAEb+D	500	189.44±8.7	292.56±12.6^a,b^	263.31±9.2^a,b^	223.85±6.8^a,b^
SOEa+D	250	173.8±13.7^c^	271.82±14.2^a,b^	194.42±12.5^a,b,c^	220.4±11.7^a,b^
Ac+D	20	161.43 ± 7.6^b^	244.54 ± 10.8^a,b^	183.34 ±7.6^a,b^	165.82 ± 6.5^b^

NC: normal control, DC: diabetic control, ASEa+D and ASEb+D: diabetic rats treated with 250 and 500 mg kg^−1^ BW of *Allium sativum *bulbs methanolic extracts respectively, AAEa+D and AAEb+D: diabetic rats treated with 250 and 500 mg kg^−1^ BW of *Allium ascalonicum *bulbs methanolic extracts respectively, SOEa+D: diabetic rats treated with 250 mg kg^−1^ BW of *Salvia officinalis *leaves methanolic extract, Ac+D: diabetic rats treated with acarbose. Each value is the mean ± SD. of ten separate experiments.a p < 0.05 vs. Time0, b p < 0.05 vs. DC, c p > 0.1 vs. Ac+D.

*Effects of ASE, AAE and SOE on PBG in long term treatment*

The levels of PBG in alloxan-diabetic rats are given in Table 3. Daily administration of methanolic plants extracts reduced PBG concentration in all groups at the end of first, second and third weeks. PBG concentrations decreased 37 % in ASEa+D, 36 % in ASEb+D, 13 % in AAEa +D, 22 % AAEb +D and 32 % in SOEa+D groups, (p < 0.05 vs. time 0). Moreover in Met+D group PBG levels dropped off by 37 % (p < 0.05 vs.time0). After 3 weeks of treatment, the hypoglycemic outcome of ASEa+D, ASEb+D and SOEa+D show similar effects to that of Met+D group (p > 0. 1). These results indicate that after long term treatment, all groups showed significant effects in reducing PBG levels comparing to DC group (p < 0.05). 

**Table 3:** Glycemic control by ASE, AAE and SOE in alloxan-diabetic rats after 3 weeks treatment. PBG was measured at the end of first, second and third weeks of the experimental period.

**Groups**	**Dose** **(mg kg** ^−1^ **BW)**	**Postprandial blood glucose (mg/dL)**			
		**Days after a single dose daily treatment **			
		**0**	**7**	**14**	**21**
NC	-	79.33±16.19^b^	85.5±5.26^b^	70.5±8.7^b^	75±5.16^b^
DC	-	303.23±12.7	309.31±14.2	317.33±8.8	324.62±12.3^a^
ASEa+D	250	287.25±7.8^b^	234.76±6.3^a,b,c^	207.11±10.2^a,b,c^	182.67±6.4^a,b,c^
ASEb+D	500	294.56±8.4	239.52±7.5^a,b,c^	204.73±9.2^a,b,c^	186.65±8.2^a,b,c^
AAEa+D	250	291.32±11.6	276.51±9.7^a,b^	255.2±8.4^a,b^	253.74±10.2^a,b^
AAEb+D	500	284.72±9.2^b^	254.2±6.2^a,b,c^	215.65±8.9^a,b,c^	221.42±5.3^a,b^
SOEa+D	250	294.67±11	239.1±8.7^a,b,c^	228.31±15.6^a,b,c^	200.84±10.5^a,b,c^
SOEb+D	500	-	-	-	-
Met+D	20	308.28 ± 10.2	257.3 ± 7.9^a,b^	218.4 ± 9.3^a,b^	195.4 ± 6.2^a,b^

NC: normal control, DC: diabetic control, ASEa+D and ASEb+D: diabetic rats treated with 250 and 500 mg kg^−1 ^BW of *Allium sativum *bulbs methanolic extracts respectively, AAEa+D and AAEb+D: diabetic rats treated with 250 and 500 mg kg^−1^ BW of *Allium ascalonicum *bulbs methanolic extracts respectively, SOEa+D and SOEb+D: diabetic rats treated with 250 and 500 mg kg^−1 ^BW of *Salvia officinalis *leaves methanolic extracts respectively, Met+D: Diabetic rats treated with metformin. Each value is the mean ± SD. of ten separate experiments.

^a^ p < 0.05 vs. Time 0, ^b^ p < 0.05 vs. DC, ^c^ p > 0.1 vs. met+D

*Effects of ASE, AAE and SOE on Ins and Glut-4 genes expression*

**Figure 1:**
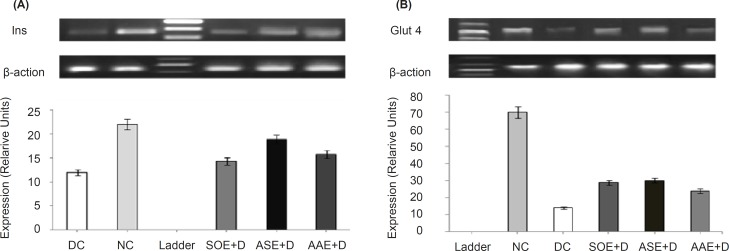
Changes in insulin and cardiac glucose transporter-4 mRNAs expression profiles in rats. ASE, AAE (500 mg kg^−1^ BW) and SOE (250 mg kg^−1^ BW) were orally administered once daily for 3 weeks to ASEb+D, AAEb +D and SOEa+D groups and vehicle to NC and DC groups. Total mRNAs were separately prepared from the individual pancreases (*Ins*) and heart left ventricle (*Glut-4*) of the rats. The relative levels of specific mRNAs were assessed by RT-PCR and images of radiographs were analyzed with TotalLab v1.10 from Phoretix using 1D analysis. Background was subtracted using the rolling disc method with a radius of 200 ???, and density was measured as pixel intensity. (A) Analysis of *Ins *transcripts (186 bp) in pancreas tissue in ASE, AAE and SOE treated diabetic rats, showed elevated levels of *Ins *transcripts compared with DC group (p < 0.05). Densitometric scanning revealed increase in INS gene transcripts, 0.57-fold (p < 0.05) by ASE, 0.31 fold (p < 0.05) by AAE and 0.19-fold (p < 0.05) by SOE respectively, as compared to DC group. (B) Analysis of *Glut-4 *transcripts (449 bp) in heart tissue in ASE, AAE and SOE treated diabetic rats showed elevated levels of *Glut-4 *transcripts compared with DC group (p < 0.05). Densitometric scanning revealed increase in *Glut-4 *gene transcripts, 1.21-fold (p < 0.05) by ASE, 0.71-fold (p < 0.05) by AAE and 1.05-fold (p < 0.05) by SOE as compared to DC group. The data represent the average of three or four samples (only one image was shown here). *β*-actin was used as internal control. NC, normal control; DC, diabetic control; ASE+D, diabetic rats treated with *Allium sativum *bulbs methanolic extract (500 mg kg^−1^ BW); AAE+D, diabetic rats treated with *Allium ascalonicum *bulbs methanolic extract (500 mg kg^−1^ BW); SOE+D, diabetic rats treated with *Salvia officinalis *leaves methanolic extract (250 mg kg^−1^ BW)

Gel electrophoresis images reveal elevated expression of both *Ins *(Figure 1A) and *Glut-4 *(Figure 1B) transcripts by ASE (500 mg kg^−1^ BW)*, *AAE (500 mg kg^−1^ BW) and SOE (250 mg kg^−1^ BW). Also, densitometric scanning reveals increase in *Ins *and *Glut-4 *genes transcripts by 0.57-fold (p < 0. 05) and 1.21-fold (p < 0.05) by ASE, 0.31 fold (p < 0.05) and 0.71-fold (p < 0.05) by AAE and 0.19-fold (p < 0.05) and 1.05-fold (p < 0.05) by SOE respectively, as compared to DC group. In these assays, *β*-actin gene was used as internal control.

*Effect of ASE, AAE and SOE on rat intestinal sucrase and maltase activities*

The methanolic extract of *Salvia of*fi*cinalis L. *leaves showed strong inhibitory activity for both sucrase and maltase by 48.05 % (±2.87) and 49.21 % (±2.28) respectively. Methanolic extracts of *Allium sativum *and *Allium ascalonicum *bulbs exhibited inhibitory activities for sucrase in order of, 24.11 %( ±1.45) and 17.41 %( ±2.81) and for maltase in order of, 19.37 % (±1.81) and 14.62 % (±2.12).

## Discussion

The stimulation of experimental diabetes in rat, using chemicals that selectively destroy pancreatic *β*-cells is very convenient and simple to use. The most usual substances to induce diabetes in rats are alloxan monohydrate and streptozotocin. The effects of alloxan on pancreatic *β*-cells consist of several processes such as oxidation of essential-SH groups, inhibition of glucokinase, generation of free radicals and disturbances in intracellular calcium homeostasis (33). Alloxan has been observed to cause a massive reduction of the *β*-cells of the islets of Langerhans and induce hyperglycemia (34). In this study, we have observed that *Allium sativum*, *Allium ascalonicum *and *Salvia officinalis *methanolic extracts decrease blood glucose level in alloxan diabetic rats. The possible mechanism of action of plants extracts could be correlated with the plasma insulin levels (35-36).

It is generally accepted that glibenclamide (a sulfonylurea family drug) causes reduction of blood glucose predominantly via stimulation of insulin release from pancreatic *β*-cells. Additionally, during long-term treatment, an insulin-independent blood glucose-decreasing mechanism may operate (37). Acarbose, an alpha-D-glucosidase inhibitor (38-39), reversibly inhibits alpha-glucosidases that exist in the brush-border of the small intestinal mucosa (40). Acarbose can inhibit maltose absorption through the inhibition of maltase and alpha-amylase (41). 

The present study demonstrates that in short term model, oral administration of ASE and AAE have hypoglycemic action like glibenclamide while SOE acts like acarbose. Previous studies reveal that ASE and its sulfide components like diallyl trisulfide and allicin increase plasma insulin level and insulin sensitivity (42-43). Moreover, total content of phenolic and diallyl disulfide compounds in AAE are higher than in ASE (44). So the hypoglycemic effects of ASE and AAE may be predominantly due to increase in insulin secretion or (and) insulin sensitivity. Our results show that ASE and AAE have inhibitory effects on intestinal maltase and sucrase . This α-glucosidase inhibition property have important role in antihyperglycemic action of ASE and AAE.

In a dose dependent manner, similar to acarbose, SOE reduced PBG in short term period. Our results show that SOE had strong inhibitory effects on intestinal sucrase and maltase activities. It is known that rosmarinic acid and other phenolic components of *Salvia officinalis *have α-glucosidase inhibitiory activity (45). 

Metformin, a biguanide, which is considered as an insulin sensitizer (46), reduces fasting plasma glucose concentrations by reducing rates of hepatic glucose production (47). Based on our results, in the long-term period, ASE and SOE reduce PBG similar to meformin. In addition to other possible hypoglycemic mechanisms, flavonoids may play important roles in reducing PBG. Some flavonoids such as quercetin and catechin can inhibit glucokinase and glucose 6-phosphatase, the enzymes that control gluconeogenesis (48), and increase the storage of glucose in the liver with reduce glycogen breakdown (49). ASE and AAE are rich in flavonoids such as quercetin 3,4-diglucoside and quercetin 4-glucoside (50). SOE is rich in kaempferol, luteolin and quercetin (17). Additionally, essential oil from sage increases hepatocyte sensitivity to insulin and inhibits gluconeogenesis (51). Altogther, in long-term treatment, ASE and SOE may act by similar hypoglycemic mechanisms as metformin. 

OGTT results indicate that the control of PBG level by SOE is possibly mediated by the regulation of glucose uptake from the intestinal lumen, through the inhibition of carbohydrate digestion or absorption (52), resembling the acarbose effect. In order to support this suggestion, our results show that SOE has a strong inhibitory effect on both sucrase and maltase activities. Also, ASE and AAE inhibitory effects on sucrase and maltase activities may be responsible for the control of PBG level. 

A primary metabolic effect of insulin is to stimulate the uptake of circulating glucose into muscle and adipose tissue. Conceptually, insulin-mediated glucose transport could occur by either stimulating the activity of existing cell surface glucose transport proteins or by translocation of an intra-cellular transporter to the cell surface in response to insulin. We now know that insulin-stimulated glucose uptake is mediated by the muscle and fat specific, insulin regulatable glucose transporter iso-type 4 (Glut4). Our results demonstrate that ASE, AAE and SOE enhance *Ins *and *Glut-4 *mRNA levels in heart muscle. Enhancement in *Ins *transcripts may be related to the stimulation of the surviving beta cells. Up regulation of *Glut-4 *expression, an insulin-dependent GLUT, might be an effect of *Ins *improvement and could cause upgrading in the utilization of blood glucose by muscles. In according to our results, enhancement in expression of *Ins *and *Glut-4 *genes may mediate hypoglycemic effects of the plants extracts. Our study is the first to report the up-regulation of *Ins *and *Glut-4 *genes expression by the ASE, AAE and SOE in diabetic Wistar rats. More researches are needed to prove the effects of these plants extracts on the enhancement of *Ins *and *Glut-4 *genes expression.

In conclusion, in addition to the different mechanisms by which the methanolic extracts of *Allium sativum, Allium ascalonicum *and *Salvia officinalis *exert their antihyperglycemic actions, up regulation of *Ins *and *Glut-4 *genes expression and inhibition of *α*-glucosidases activities may play a considerable role in this process. 
